# Interaction of NDRG1 and MRE11 Modulates DNA Replication and Repair

**DOI:** 10.3390/cancers18081303

**Published:** 2026-04-20

**Authors:** Hanna M. Doh, Nina Kozlova, Zhipeng A. Wang, Hwan Bae, Philip A. Cole, Taru Muranen

**Affiliations:** 1 Department of Medicine, Beth Israel Deaconess Medical Center, Boston, MA 02215, USA; hanna.doh@gmail.com (H.M.D.);; 2 BIDMC Cancer Center, BIDMC Cancer Research Institute, Boston, MA 02215, USA; 3 Biological and Biomedical Science PhD Program, Harvard Medical School, Boston, MA 02115, USA; 4 Department of Biological Chemistry and Molecular Pharmacology, Harvard Medical School, Boston, MA 02115, USA; zaw29@med.miami.edu (Z.A.W.); pacole@bwh.harvard.edu (P.A.C.); 5 Division of Genetics, Departments of Medicine and Biological Chemistry and Molecular Pharmacology, Brigham and Women’s Hospital, Boston, MA 02115, USA; 6 Desai Sethi Urology Institute & Sylvester Comprehensive Cancer Center, University of Miami Miller School of Medicine, Miami, FL 33136, USA; 7 Department of Medicine, Harvard Medical School, Boston, MA 02115, USA

**Keywords:** pancreatic cancer, DNA repair, DNA replication, NDRG1, MRE11, MRN complex, drug resistance

## Abstract

**Background/Objectives**: Pancreatic ductal adenocarcinoma (PDAC) is a lethal disease with limited treatment options. Patients are treated with DNA damaging chemotherapies which act by inducing DNA damage in rapidly dividing tumor cells. Unfortunately, these tumors frequently develop treatment resistance, underscoring the need to understand resistance mechanisms in order to develop better treatment strategies. DNA damage response (DDR) detects and repairs DNA damage, and the DDR pathway has been shown to contribute to chemoresistance. Another factor known to drive chemoresistance in PDAC is the dense stroma, composed of extracellular matrix proteins secreted by cancer-associated fibroblasts (CAFs). Our recent work identified a CAF-induced resistance mechanism involving N-myc downstream regulated gene 1 (NDRG1). CAF-induced signaling resulted in the phosphorylation of NDRG1 and NDRG1-dependent DNA repair and protection from chemotherapies. Loss of NDRG1 resulted in increased chemotherapy-induced DNA damage and decreased replication fork speed and recovery. **Methods**: To gain insight into the molecular mechanism of NDRG1-mediated DNA repair and replication, we performed a BioID screen to identify binding partners of NDRG1. We further assessed the mechanistic roles of the identified interaction partners on DNA repair using DNA replication and repair assays such as the Comet assay and DNA fiber assays. **Results**: Our BioID screen identified meiotic recombination 11 (MRE11) protein, a nuclease involved in DDR, as a putative NDRG1 interacting protein. Interaction between MRE11 and NDRG1 was enriched during the late S/early G2 cell cycle phases and under replication stress. However, this interaction is likely indirect as the interaction only occurred in a cellular context and not with in vitro purified proteins. Blocking NDRG1 phosphorylation or blocking MRE11 exonuclease activity both resulted in protection of newly synthesized DNA at stalled replication forks. In NDRG1 knockout cells, blocking MRE11 led to decreased protection of nascent DNA, suggesting that NDRG1 and MRE11 may be acting in the same pathway and that NDRG1 is required for MRE11’s activity at stalled forks. **Conclusions**: In summary, our work has uncovered a protein complex between NDRG1 and MRE11 that may play a key role in chemoresistance due to its role in the processing of stalled replication forks.

## Simple Summary

Chemotherapies work by inflicting DNA damage, and cancer cells rely on effective DNA repair to become treatment resistant. Targeting DNA repair programs in rapidly growing tumor cells could provide novel therapeutic targets. This work has identified a protein complex between two DNA repair proteins, NDRG1 and MRE11. This interaction is increased in the G2/S phase of the cell cycle and protects nascent DNA from degradation at stalled replication forks, contributing to DNA replication and repair.

## 1. Introduction

Pancreatic ductal adenocarcinoma (PDAC) is a devastating disease with a 5-year overall survival rate of ~13% [1,2]. One of the main reasons for the high mortality rate of PDAC is that tumors frequently develop chemoresistance. Alterations in DNA damage response (DDR) pathways have been linked to chemoresistance, and a large subset of PDAC patients display genetic alterations in DDR pathways [3]. Despite the role of DDR in chemoresistance, the molecular mechanisms that regulate DDR pathways and drive chemoresistance in PDAC have not been fully elucidated.

A key driver of chemoresistance in PDAC is the dense tumor microenvironment (TME), which consists of a variety of non-tumor cell types including cancer associated fibroblasts (CAFs). CAFs are known for providing support to cancer cells, thus protecting cancer cells from chemotherapies [4]. Our lab recently identified a novel molecular mechanism of CAF-induced resistance to chemotherapies in PDAC involving the abundant secretion of extracellular matrix proteins, leading to the phosphorylation of a newly identified DNA repair protein, N-myc downstream regulated gene 1 (NDRG1), inside tumor cells [5]. *NDRG1* is a stress response gene known to function in in various cellular processes and its exact role in cancer remains highly controversial with studies reporting both pro-tumorigenic and tumor-suppressive functions. Recently, there have been studies linking NDRG1 to the DNA damage response and chemotherapy resistance in cancer. For example, in glioblastoma, NDRG1 was shown to mediate resistance towards alkylating chemotherapy by removing O6-methylguanine adducts [6]. In non-small cell lung cancer (NSCLC), NDRG1 was implicated in mediating drug resistance towards platinum-based chemotherapy through activation of the nucleotide excision repair (NER) pathway [7]. NDRG1 has also been shown to facilitate DNA replication in the context of Kaposi’s sarcoma-associated herpesvirus (KSHV) viral genome maintenance through interacting with proliferating cell nuclear antigen (PCNA) [8]. Our study was the first to investigate a novel role for NDRG1 in resolving chemotherapy-induced replication stress in PDAC. Specifically, we found that phospho-NDRG1 physically associated with replication forks, and played a role in resolving stalled replication forks by reducing R-loops [5]. NDRG1 has been shown to interact with other proteins and these protein-protein interactions regulate various cellular processes relevant to tumor growth including DNA repair [6,9,10]. Furthermore, we have recently identified and characterized a novel interaction between NDRG1 and transglutaminase 2 (TGM2), which modulated replication fork stability and recovery [11]. In an effort to uncover further mechanistic details underlying NDRG1-mediated chemoresistance and DNA repair in PDAC, we set out to identify new NDRG1 binding partners.

This research identified a putative new NDRG1 binding partner, meiotic recombination 11 (MRE11). MRE11 is a member of the MRE11-RAD50-NBS1 (MRN) complex, and it is required for key DNA repair pathways that resolve chemotherapy-induced DNA damage including homologous recombination (HR) repair of double-stranded breaks (DSBs), and restart of stalled or collapsed replication forks [12]. MRE11 is highly expressed in PDAC tumors, and its high expression correlates with poor overall survival. Under conditions of replication stress, the MRN complex is rapidly recruited to stalled forks through interactions with phospho-replication protein A (RPA) and poly (ADP-ribose) polymerase 1 (PARP1) [13,14]. Once recruited to stalled forks, MRE11 nuclease activity functions to degrade nascent DNA at stalled forks, creating ssDNA tracts required to activate recombination and repair pathways, ultimately promoting fork restart [15]. However, this MRE11-mediated resection is tightly controlled by factors such as BRCA2, PARP1, and RAD51, as extensive MRE11-dependent degradation of stalled forks can be deleterious and lead to chromosomal instability [16,17]. The importance of MRE11-mediated resection of stalled forks for cell survival is underscored by studies showing that knockdown of MRE11 reduces growth in cells depleted of PARP1 or breast cancer gene 2 (BRCA2) [15]. MRE11-dependent DNA end resection has recently been shown to contribute to PARP inhibitor resistance (for the treatment of BRCA1/2 mutant PDAC) and gemcitabine, both drugs used in the treatment of PDAC [18]. Interestingly, studies in yeast have also revealed that the closely related MRX (Mre11-Rad50-Xrs2) complex promotes fork restart at stalled forks in a nuclease-independent manner, by tethering sister chromatids together [19,20]. These studies collectively reveal that the MRN complex utilizes both its enzymatic resection function and structural tethering function to influence DDR and maintain genome integrity.

The objective of this study was to investigate how a putative protein complex involving NDRG1 and MRE11 may influence chemoresistance and stalled replication fork dynamics.

## 2. Materials and Methods

### 2.1. Cell Lines

SW1990 (CRL-2172) and SUIT2 were a kind gift from Dr. Nada Kalaany. HEK293T cells were purchased from ATCC. All cell lines were tested Mycoplasma negative by using the MycoAlert Detection Kit (Lonza, Walkerswille, MD, USA) and STR authenticated. Cells were maintained in DMEM supplemented with 10% FBS, 50 IU/mL penicillin, and 50 μg/mL streptomycin. Rosetta (DE3) competent cells were purchased from Sigma Aldrich, St Louis, MO, USA (#70954).

### 2.2. Plasmids and Cloning

To generate the NDRG1 BioID construct, the coding sequence of WT *NDRG1* [5] was subcloned into mycBioID2-pBABE-puro (kind gift from Dr. Kyle Roux Addgene #80900, no longer available on Addgene, Watertown, MA, USA) via *BamHI* and *EcoRI*, allowing for the expression of the C-terminal Myc-tagged *NDRG1*-fused to BirA (R118G). To generate the pLenti6-V5-*NDRG1* construct, the coding sequence of *NDRG1* was ordered from GenScript, subcloned into pLenti6/V5-p53_wt *p53* (Addgene #22945 [21]) by cutting out the coding sequence of *p53* and pasting the coding sequences of *NDRG1* into via *XbaI* and *SpeI* restriction sites, generating the C-terminal V5-tagged *NDRG1*-WT construct. pLenti CMV V5-Luciferase Blast construct was a gift from Dr. Eric Campeau (Addgene plasmid #21474) [22]. pET28a-TEV-*NDRG1*-FL construct was a kind gift from Dr. Venla Mustonen [23]. pFastBac1-*MRE11*-FLAG, pFastBac1-*Nbs1*, and pAceBac1-*Rad50*-His plasmids were a kind gift from Dr. Ilya Finkelstein. To generate NDRG1 KO cells, we used LentiCRISPR v2 (a gift from Feng Zhang (Addgene plasmid # 52961) [24], and the construct for *NDRG1* KO was created by annealing the following sense and antisense oligonucleotides: KO-2s CACCGGTACCCCTCCATGGATCAGC and KO-2as AAACGCTGATCCATGGAGGGGTAC, followed by subcloning in the LentiCRISPR v2 backbone.

### 2.3. Production of Lentiviruses and Stable Cell Lines

For lentiviruses, 70–80% confluent HEK-293T cells were co-transfected with psPAX2 (Addgene # 12260), pVSVg (gift from Joan Brugge), and LentiCRISPR v2 with subcloned g*NDRG1* KO-2, by Lipofectamine 3000 (Thermo Fisher, Waltham, MA, USA). Media was collected over a period of 24 to 72 h post-transfection in 24 h batches, pooled, centrifuged and filtered through a 0.45 µm filter. LentiCRISPR v2 infected cells were cultured in the presence of Puromycin (1 µg/mL) for 96 h.

### 2.4. BioID Pull-Down Experiments

*NDRG1*-myc-BioID2-pBABE2-puro construct was used for the BioID experiment, and the empty myc-BioID2 construct (Addgene #80900) was used as a control. BioID labelling and sample preparation was done as described before Roux et al., 2018 [25]. 3 × 15 cm dishes (SW1990 cells) were grown in non-conditioned or CAF-conditioned media with 0% serum, at 80% confluency. The cells were incubated with 50 µM biotin (18 h), washed twice with PBS, cells pelleted and collected. Cell lysis was done in RIPA buffer (BP-115, Boston BioProducts, Milford, MA, USA) with benzonase (E1014, Sigma Aldrich), sonicated, lysate was centrifuged at 14,000× *g* for 15 min. 1 mg of total protein was used for streptavidin-pull down using Dynabeads^TM^ MyOne^TM^ Streptavidin C1 (65001, Thermo Fisher). The lysate and beads were incubated for 16 h at +4 °C, washed and analyzed by LC-MS/MS. On-bead trypsin digestion and LC-MS/MS analysis was done at BIDMC Mass Spectrometry core. Full analysis of the BioID hits has been included as Supplementary File S1.

### 2.5. BioID SAINT Score Analysis

**Figure 1:**
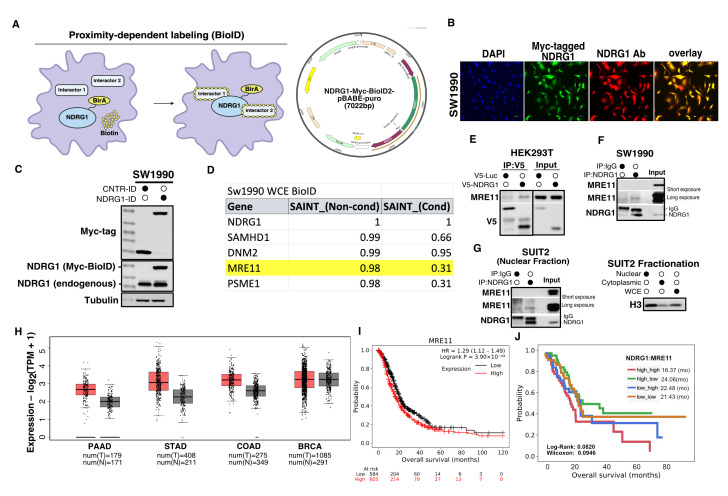
Identification of MRE11 as a potential binding partner of NDRG1. (**A**) Schematic showing the premise of proximity-dependent labeling (BioID) screen we performed in SW1990 pancreatic cancer cells and the BioID construct used expressing NDRG1-myc-BioID2 fusion protein (shown on **right**). (**B**) Immunofluorescence staining showing localization of NDRG1-myc-BioID2 in SW1990 cells (green: myc Ab, red: NDRG1 Ab, blue: DAPI). Scale bar: 100 µm. (**C**) Western blot showing expression levels of control-myc-BioID and NDRG1-myc-BioID constructs in SW1990 cells. NDRG1 blot shows endogenous NDRG1 (lower band) and the NDRG1-myc-BioID2 construct (upper band). (**D**) Table displaying five highest-scoring interaction partners from BioID screen performed in whole cell extracts (WCE) from SW1990, MRE11 is highlighted in yellow. Full list is provided as Supplementary File S1. (**E**) HEK293T cells were transfected with V5-luciferase or V5-NDRG1 as indicated by black dot, and co-immunoprecipitation was performed using V5 antibody to detect binding between V5-NDRG1 and endogenous MRE11. V5-luciferase expressing cells were used as negative control for immunoprecipitation. Input lysates are shown on **right**. (**F**) Endogenous co-immunoprecipitation with NDRG1 antibody was performed using SW1990 WCE, to detect endogenous NDRG1 interaction with MRE11. IgG rabbit control antibody was used as a negative control for immunoprecipitation. Input from lysate is shown on **right**. (**G**) Endogenous co-immunoprecipitation between NDRG1 and MRE11 was performed using SUIT2 nuclear fraction. NDRG1 Ab was used to pull down NDRG1, and IgG rabbit control antibody was used as a negative control for immunoprecipitation (**left**); blot showing the controls for the fractionation of SUIT2 cell lysate (**right**). (**H**) RNAseq expression data from The Cancer Genome Atlas (TCGA) PanCancer Atlas and Genotype-Tissue Expression (GTEx) projects were used to determine MRE11 expression in normal (grey) vs. tumor (red) samples in pancreatic cancer (PAAD), stomach adenocarcinoma (STAD), colon adenocarcinoma (COAD) and breast cancer (BRCA) [32]. (**I**) 1189 pancreatic cancer patients were split into two cohorts (high expression and low expression) based on MRE11 RNAseq data. Best cutoff was determined by using all available cutoff values between lower and upper quartiles of gene expression, computing false discovery rate (FDR) using the Benjamini-Hochberg method to correct for multiple hypothesis testing, and determining the cutoff value with the highest significance (lowest FDR) [33]. (**J**) Pancreatic cancer patients from the TCGA Pan-Cancer data set were stratified into four high/low expression groups based on mRNA expression levels of MRE11 and NDRG1 (relative to the median values within the cohort) and subjected to survival analysis. Significance was determined using Log-Rank and Wilcoxon tests. The uncropped blots are shown in Supplementary Materials.

The SAINT (Significance Analysis of INTeractome) analysis of the BioID hits was done with the The Contaminant Repository for Affinity Purification (CRAPome) as described in [26,27]. *H.Sapiens* was used as the reference organism, proximity-dependent biotinylation as the experiment type, and spectral counts (SPC) as quantitation type. Top scoring targets are presented in Figure 1D and the full list presented in Supplemental Data.

### 2.6. Western Blot

RIPA buffer (BP-115, Boston Bioproducts) was used for cell lysis, complete protease inhibitor cocktail tablet (Roche, Indianapolis, IN, USA) was included. Cells were sonicated and centrifuged (14,000× *g*, 10 min, +4 °C). Proteins (~20 μg/sample) were separated on 4–12% pre-cast Tris-Glycine gels (Invitrogen, Carlsbad, CA, USA) and transferred to nitrocellulose membranes. The membranes were blocked in 5% milk in Tris-buffered saline with Tween 20, followed by incubation in primary antibodies. Antibodies used: NDRG1 (#9485S, Cell signaling technologies (CST), Beverly, MA, USA), pNDRG1 Thr346 (#5482, CST), V5 (#R96025, Thermo Fisher), MRE11 (#4895, CST), Vinculin (#13901, CST), Myc (#2276, CST), Tubulin (#ab7291, Abcam, Waltham, MA, USA), H3 (#4499, CST), Cyclin A2 (#), pCDK2 (#2561, CST) Appropriate secondary antibodies (peroxidase-conjugated IgG, CST) were used. The ECL Kits (Thermo Fisher) were used for signal detection. The Westerns were imaged by Amersham Imager 600. Images were imported to ImageLab software 6.1 (Biorad, Hercules, CA, USA). All uncropped Western blots have been included in the Supplementary File S2.

### 2.7. Co-Immunoprecipitation

Whole cell extract IPs: RIPA buffer (BP-115, Boston Bioproducts) was used with containing complete protease inhibitor cocktail tablet (Roche). Cells were sonicated and centrifuged (14,000× *g*, 10 min, +4 °C). Nuclear fractions: Cells were incubated in hypotonic buffer (20 mM Tris-HCL pH 8.3, 10 mM NaCl, 3 mM MgCl_2_, protease inhibitor tablets) on ice, 10 min after which 10% NP40 was added. Cells were vortexed and centrifuged to isolate cytoplasmic fraction (supernatant). Nuclear pellet was suspended in RIPA, sonicated, and centrifuged. For V5 IP, 500 mg protein per sample was incubated with 2 µL of V5 antibody (#R96025, Thermo Fisher) (2 h, +4 °C, rotating). For anti-NDRG1 IP samples, 500 mg protein/sample was incubated with NDRG1 (#9485S, CST), or IgG Rabbit negative control (#2729, CST) antibodies for 2 h at +4 °C. 60 µL of Protein A/G beads (#78609, Thermo Fisher) were used/sample and incubated overnight at +4 °C. Beads were washed 3× with RIPA buffer, beads boiled in 2× SDS loading buffer. Samples were run on 4–12% pre-cast Tris-Glycine gels (Invitrogen) and Western blotted.

### 2.8. Immunofluorescence

Cells were fixed with 4% PFA (15 min, RT), permeabilized with 0.1% Triton-X 100 in PBS for 10 min at RT. Coverslips were blocked in 5% BSA (BP1600-100, Thermo Fisher) for 1 h at RT. Coverslips were incubated with primary antibodies at +4 °C mixed in antibody dilution buffer (1% BSA, 0.3% Triton X-100 in PBS) overnight. Primary antibodies: rabbit NDRG1 (1:100, CST), mouse Myc-tag antibody (#2276, CST). Next, coverslips were washed 3× in PBS and incubated with secondary antibodies (1 hr, RT). Secondary antibodies: Alexa-Fluor 488 goat anti-mouse IgG (A11029, Invitrogen), Alexa-Fluor 546 goat anti-rabbit IgG (A11010, Invitrogen). Finally samples were washed 3× in PBS, incubated with Hoechst (1:5000, Sigma-Aldrich) for 10 min RT. Image aquisition was done with Zeiss LSM 880 Confocal (White Plaines, NY, USA), with 63× PlanApo objective or with Keyence BZ-X800 microscope (Itasca, IL, USA).

### 2.9. Proximity Ligation Assay

For the PLA Duolink PLA Kit (DUO92102, Sigma-Aldrich) was used following the manufacturers protocol. Cells were fixed with 4% PFA for 15 min at RT, permeabilized in 0.1% Triton-X 100 in PBS for 15 min at RT. Samples were blocked in Duolink Blocking Buffer and incubated with the rabbit NDRG1 antibody (1:100, CST) and mouse MRE11 antibody (1:100 ab214, Abcam) overnight at +4 °C. Next, cells were incubated with Duolink probes, ligation solution, and amplification solution. After the final washes, cells were incubated with Phalloidin (#A12379, Invitrogen) for 40 min RT. Cells were counterstained with Hoechst (1:5000) for 10 min RT. Image acquisition was done with Zeiss LSM 880 Upright Confocal System, or with Keyence BZ-X800 microscope. Confocal images were imaged as a Z-stack, processed using ‘maximum intensity projection’ tool provided by the Zen 2009 software. Cell Profiler 4.2.8 (https://cellprofiler.org/) was used for PLA foci quantification with speckle analysis pipeline [28].

### 2.10. AlphaFold Prediction

AlphaFold prediction was done using Cosmic^2^ [29]. Human full length (1-394) NDRG1 (#Q92597) and full length (1-707) MRE11 (#F8W7U8) protein sequences were uploaded to Cosmic^2^. AlphaFold2 tool was selected, and we used the full_dbs database and multimer model. Output models were analyzed with PyMol 3.1 software (https://www.pymol.org/).

### 2.11. Chemicals, Reagents and Drugs

Biochemicals and enzymes were of analytic grade and were purchased from commercial suppliers. Gemcitabine (S1714, Selleck chemicals, Houston, TX, USA) was reconstituted in DMSO and used at a final concentration of 1 µM. HU (H8627, Sigma-Aldrich) was reconstituted in DMEM used at a concentration of 2 mM. 5-chloro-2′-deoxyuridine (CldU) (C6891), 5-iodo-2′-deoxyuridine (IdU) (I7125), DAPI (D9542), Thymidine (T1895), and HT-DNA (D6898) were from Sigma. Restriction enzymes and Lambda Phosphatase (P0753S) were from New England Biolabs (NEB), Ipswich, MA, USA. Mirin (#HY-19959, Medchem Express, Monmouth Junction, NJ, USA) was reconstituted in DMEM and used at indicated concentrations. Small molecule SGK1 inhibitor BLU6340 was made by Blueprint Medicines, Cambridge, MA, USA. MK2206 and CHIR-99021 were purchased from Selleck chemicals and reconstituted in DMSO and used at indicated concentrations.

### 2.12. DNA Fiber Fork Protection Assay

DNA fibers were performed as described previously [30]. Briefly, cells were sequentially pulsed using thymidine analogues 50 µM CIdU (Sigma, C6891) and IdU 150 µM (Sigma, 17125) for 30 min for each pulse with 2× PBS washes in between. Following the thymidine analogue pulses, cells were treated with 2 mM HU for 5 h in the presence or absence of inhibitors. Cells were trypsinized and resuspended in PBS. 2.5 µL of cell suspension was added on SuperFrost plus slide (#48311-703, VWR, Radner, PA, USA) for 4 min. Next, 7.5 µL spreading buffer (0.5% SDS, 200 mM Tris-HCl pH 7.4, 0.5 mM EDTA) was added for 2 min. After the incubation, the slides were tilted at 15 degrees. Next, the slides were air dried and fixed in 3:1 methanol:acetic acid solution for 2 min, followed by 2.5 M HCl incubation (30 min), and 3% BSA/PBST block for 1 h. Primary antibodies used were anti-CIdU (ab6326 Abcam, 1:100) and anti-IdU (BD-347580, 1:20), 1 h incubation. After 3× washes with PBS, fibers were stained with secondary Alexa-Fluor conjugated antibodies (30 min), washed, air-dried and mounted. Slides were imaged with Zeiss LSM 880 Confocal. Measurement of replication structures was done manually using Fiji x86-64 program (https://imagej.net/software/fiji/). Pooled data from at least *n* = 2 biological replicates are shown.

### 2.13. Drug Treatments and IC50 Calculations

Cells were plated onto 96 well plates (7.5 × 10^3^ per well), the next day, gemcitabine and mirin were added using a digital dispenser (Tecan D300e), Corvallis, OR, USA. For gemcitabine, concentrations from a log 1/3.16 step down from the highest indicated concentration was used. 72 h after, cell viability was analyzed using Presto Blue HS (Invitrogen). Values are normalized to DMSO-treated control wells and analyzed in GraphPad Prism 10. Non-linear regression analysis was used to fit the data to log(drug) vs. response (variable slope) curve. IC50 values for each treatment are shown on the graph.

### 2.14. Protein Purification

NDRG1 purification was performed as described in Mustonen et al. [23]. Briefly, pET28a-TEV-*NDRG1*-FL (a kind gift from Dr. Venla Mustonen), was transformed into Rosetta (DE3) competent cells. Cells were cultured in autoinducing ZYM-5052 medium, supplemented with 50 µg/mL kanamycin and 34 µg/mL chloramphenicol for 4 h at 37 °C and for 16 h at 20 °C. The cells were collected by centrifugation at 5020× *g* for 40 min at 4 °C. Cell pellets were resuspended in cold buffer A (50 mM HEPES, pH 7.0, 500 mM NaCl, 10 mM imidazole, pH 7.0, and 0.25 mM TCEP, pH 7.0), with protease inhibitor cocktail tablet (Roche), and French press was used to lyse bacterial cells. The supernatant was incubated with HisPur^TM^ Ni-NTA Resin (#88222, Thermo Fisher) that was pre-equilibrated with buffer A. Resin was loaded onto a column and washed with buffer A containing 50 mM imidazole. The proteins were eluted using buffer A with 250 mM imidazole. Eluted proteins were concentrated using 30 kDa Amicon Ultra-15 Centrifugal Filters (#UFC900324, Thermo Fisher) and loaded onto Superdex75 10/300 column equilibrated with buffer A. Elution fractions containing the desired His-NDRG1 proteins were collected and concentrated using 30 kDa Amicon Ultra-15 Centrifugal Filters.

MRN complex purification was performed as described in Myler et al. [31]. Briefly, pFastBac1-*MRE11*-FLAG, pFastBac1-*Nbs1*, and pAceBac1-*Rad50*-His constructs (kind gifts from Dr. Ilya Finkelstein), were transformed into DH10Bac cells and bacmids for each individual construct were purified. Sf21 insect cells grown in 30 °C on 15 cm dishes were used to generate baculoviruses and express the MRN complex. To generate baculoviruses, Sf21 insect cells were transfected with individual bacmids for Mre11-FLAG, Nbs1, and Rad50-His using Cellfectin II. After 72 h of virus production, the first preparation of baculoviruses for each bacmid were collected and filtered using 45 µm filter. Sf21 insect cells were transduced individually with first baculovirus preparations for Mre11-FLAG, Nbs1, and Rad50-His for 72 h to amplify the baculoviruses, and this process was repeated to obtain the second amplification of baculoviruses for each individual MRN complex component. Second amplification baculoviruses were used to infect Sf21 cells (60× 15 cm dishes) for protein production, and cells were simultaneously infected with all three baculoviruses to generate the complete MRN complex. After 72 h of infection, insect cells were harvested via scraping and collected via centrifugation at 1500× *g* for 10 min. Pellets were washed with PBS and centrifuged again at 1500× *g* for 10 min before proceeding with purification of MRN complex. Pellets were resuspended in MRN lysis buffer (50 mM KH_2_PO_4_, pH 7.4, 500 mM KCl, 2.5 mM imidazole, 20 mM β-mercaptoethanol (β-ME), 10% glycerol, 0.5% Tween-20, 1 mM phenylmethane sulfonyl fluoride (PMSF)). Pellets were sonicated and centrifuged at 100,000× *g* for 1 h at °C, and supernatant was incubated with HisPur^TM^ Ni-NTA Resin (#88222, Thermo Fisher) that was pre-equilibrated with MRN lysis buffer, for 1 h at 4 °C. Samples were centrifuged at 1500× *g* for 3 min, and Ni-NTA resin was washed with MRN lysis buffer, and MRN complex was eluted from Ni-NTA beads using buffer containing 250 mM imidazole. Eluted proteins were concentrated using 100 kDa Amicon Ultra-15 Centrifugal Filters (#UFC910024, Thermo Fisher) and loaded onto a Superose 6 column for size exclusion chromatography. Elution fractions containing the desired FLAG-His-MRN complex were collected and concentrated using 100 kDa Amicon Ultra-15 Centrifugal Filters.

### 2.15. Binding Assays with Purified Proteins

For in vitro pulldowns, 50 µg purified His-NDRG1 and 50 µg purified FLAG-His-MRN complex were added to 400 µL RIPA buffer (BP-115, Boston Bioproducts) or base buffer (10 mM Tris HCl, 100 mM NaCl, 10% glycerol, 0.01% NP40, 1 mM dithiothreitol) containing complete protease inhibitor cocktail tablet (Roche). DNA substrates were used at the following concentrations: 10 nM dsDNA 90 bp, 500 ng HT-DNA (D6898, Sigma Aldrich). Purified proteins were incubated at 4 °C for 2.5 h on rotator. 25 µL anti-FLAG M2 Affinity Gel (A2220, Sigma Aldrich) was added to mixture of purified proteins and incubated at 4 °C for 1 h on rotator. Samples were spun down at 1000× *g* for 5 min and washed with RIPA or base buffer 3 times. Proteins were eluted from beads by resuspending in 2× SDS loading buffer and boiling for 8 min. Western blot was performed to detect binding of His-NDRG1 to FLAG-MRN complex.

For semi-endogenous pulldowns using cell lysate and purified proteins as bait, cells were lysed in RIPA buffer (BP-115, Boston Bioproducts) containing complete protease inhibitor cocktail tablet (Roche). Cells were then sonicated and centrifuged at 14,000× *g* for 10 min at +4 °C and 500 mg protein per sample was incubated with 50 µg purified His-NDRG1 or 50 µg purified FLAG-His-MRN complex for 2.5 h rotating at 4 °C. 25 µL anti-FLAG M2 Affinity Gel (A2220, Sigma Aldrich) or HisPurTM Ni-NTA Resin (#88222, Thermo Fisher) was then added to mixture of purified proteins and lysates and incubated at 4 °C for 1 h on rotator. Samples were spun down at 1000× *g* for 5 min and washed with RIPA 3 times. Proteins were eluted from beads by resuspending in 2× SDS loading buffer and boiling for 8 min. Western blot was performed to detect binding of endogenous NDRG1 to purified FLAG-MRN complex, or binding of MRE11 from cell lysates to purified His-NDRG1.

### 2.16. In Vitro Phosphorylation

NDRG1 in vitro phosphorylation was performed in base buffer (10 mM Tris HCl, 100 mM NaCl, 10% glycerol, 0.01% NP40, 1 mM dithiothreitol) with 5 mM MgCl2 and 5 mM ATP. 0.5 µg NDRG1 was mixed with 0.02 µg recombinant SGK1 (ab60883, Abcam), in total reaction volume of 10 µL. Reaction was incubated at 30 °C for 1 h and then transferred to ice.

### 2.17. Statistical Analysis

GraphPad PRISM 7 was used for statistical and visual analyses. Pancreatic adenocarcinoma tumor RNAseq data (PAAD) was extracted from the TCGA pan cancer normalized data set (data_mrna_seq_v2_rsem.txt) and subjected to survival analysis in JMP statistical software (v17). Patients were stratified into high and low MRE11 and NDRG1 expression groups based on the median values within the PAAD samples. For all figures sample size and error bars are reported in the figure legends. *p*-values less than 0.05 were considered significant. Exact *p* values are shown where possible. For simple comparison of means, data were first checked for normality; if distributed normally data were then analyzed with paired or unpaired two tailed Student *t* test. If not normally distributed, data were instead analyzed with paired Wilcoxon Rank Sum (also known as Mann–Whitney U) test. The number of times experiments were performed and the number of the cells analyzed are indicated in each legend.

## 3. Results

### 3.1. MRE11 Is Identified in a BioID Screen as a Potential Binding Partner of NDRG1

To elucidate mechanisms by which NDRG1 protects cancer cells from chemotherapy-induced DNA damage, we performed a proximity-dependent biotin identification (BioID) screen in a pancreatic cancer cell line (SW1990) to identify potential binding partners of NDRG1 (Figure 1A–C). Through this screen, we identified MRE11 as a putative binding partner of NDRG1 (Figure 1D). Since MRE11 has been shown to play essential roles in DSB repair and stalled fork recovery, we hypothesized that NDRG1 may influence DNA repair and chemoresistance through its interaction with MRE11. Therefore, we decided to explore the MRE11-NDRG1 interaction further. To validate the NDRG1-MRE11 interaction in cells, we performed semi-endogenous co-immunoprecipitation (co-IP) in HEK293T over-expressing V5-tagged NDRG1 and observed co-immunoprecipitation of endogenous MRE11 with V5-NDRG1 (Figure 1E). We also performed endogenous co-IPs in pancreatic cancer cell lines to validate the endogenous interaction between MRE11 and NDRG1, and were able to detect the interaction in SW1990 and SUIT2 cells (Figure 1F,G).

To assess the potential of MRE11 as a molecular target for PDAC, we examined the clinical relevance of MRE11 in PDAC patients. We found that MRE11 expression was significantly higher in tumor samples compared to normal samples in pancreatic cancer, and other common gastrointestinal cancers, including stomach and colon cancer, but not in breast cancer (Figure 1H) [32]. Furthermore, we found that high MRE11 expression is correlated with poor overall survival in PDAC patients (Figure 1I) [33] and that combined high expression of both MRE11 and NDRG1 is associated with poorer overall survival, although the difference did not reach statistical significance (Figure 1J). These data suggest the clinical relevance of MRE11 in pancreatic cancer and provided rationale to investigate the role of MRE11 in PDAC chemoresistance.

### 3.2. MRE11-NDRG1 Interaction Is Enriched During S/G2 Phase and During Replication Stress

**Figure 2:**
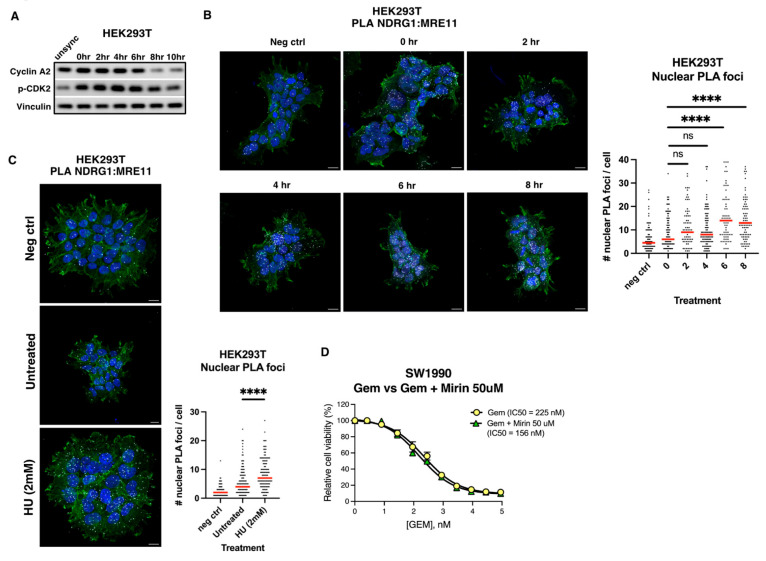
NDRG1-MRE11 complex is enriched during late S/early G2 cell cycle phases and upon hydroxyurea-induced replication stress. (**A**) HEK293T cells were synchronized via double thymidine block and western blot shows expression of cell cycle proteins (Cyclin A2, p-CDK2) to determine cell cycle phases (S vs. G2). (**B**) Proximity ligation assay (PLA) was performed on synchronized HEK293T cells at each time point after release from G1/S boundary using antibodies against NDRG1 and MRE11. As a negative control, PLA was performed using only NDRG1 antibody. (**left**) Representative images of PLA. Scale bar: 5 µm. DAPI (blue), Phalloidin (green), PLA (white); (**right**) CellProfiler was used to quantify nuclear PLA foci/cell at each time point. Significance was analyzed by two-sided Mann–Whitney test **** indicates *p* < 0.001, ns= non-significant. Red line indicates median. (**C**) PLA using antibodies against NDRG1 and MRE11 was performed in HEK293T cells treated with 2 mM HU. As a negative control, PLA was performed using only NDRG1 antibody. (**left**) Representative images of PLA. Scale bar: 5 µm. DAPI (blue), Phalloidin (green), PLA (white); (**right**) CellProfiler was used to quantify nuclear PLA foci/cell. Significance was analyzed by two-sided Mann–Whitney test **** *p* < 0.001. (**D**) Inhibition of MRE11 exonuclease activity does not sensitize cells towards gemcitabine. SW1990 cells’ IC50 response towards gemcitabine was compared in the absence or presence of 50 µM mirin. Fitted dose response curves to gemcitabine were used to generate IC50 values. Error bars are SEM corresponding to technical replicates (n = 3). The uncropped blots are shown in Supplementary Materials.

Since MRE11 is involved in HR and replication stress response, which have both been associated with resistance to DNA damaging therapies, we hypothesized that NDRG1 may regulate MRE11 function in HR or the replication stress response. HR repair of DSBs is restricted to late S/G2 cell cycle phases because HR requires the presence of a sister chromatid. Replication stress is also highest during late S phase due to common fragile sites that are difficult to replicate [34]. To determine whether the NDRG1-MRE11 interaction is enriched during cell cycle phases associated with the highest level of DSBs and replication stress, we first synchronized HEK293T cells via double thymidine block, and determined that S phase occurs from 0–6 h and G2 begins 8 h after cells are released from the double thymidine block (Figure 2A). We performed proximity ligation assay (PLA) to assess the MRE11-NDRG1 interaction at each time point after cell synchronization and interestingly, we found that the potential interaction in the nucleus was enriched at late S/G2 phases compared to G1/early S phases (Figure 2B). To further determine whether the NDRG1-MRE11 interaction might play a role in DNA repair and the replication stress response, we treated HEK293T cells with hydroxyurea, which causes replication fork stalling and replication stress, and performed PLA for NDRG1 and MRE11 (Figure 2C). We found that the NDRG1-MRE11 PLA foci were enriched in the nucleus upon treatment with hydroxyurea, further pointing to a role for the NDRG1-MRE11 interaction in DNA repair or the replication stress response (Figure 2C). However, we cannot rule out a possibility that the interaction also occurs outside of S phase.

Next, we wanted to determine whether MRE11 nuclease activity contributes to chemoresistance towards fork stalling chemotherapy in PDAC cells, given that the MRE11-NDRG1 interaction may be involved in the response to replication stress. We treated SW1990 cells with a concentration range of gemcitabine in the absence or presence of mirin, an inhibitor for MRE11 exonuclease activity. However, we did not observe a significant shift in IC50, suggesting that inhibition of MRE11 exonuclease activity does not sensitize cells towards fork stalling chemotherapies such as gemcitabine, at least in the SW1990 PDAC cell line (Figure 2D).

### 3.3. MRE11-NDRG1 Complex Formation Is Regulated by NDRG1 Phosphorylation

**Figure 3:**
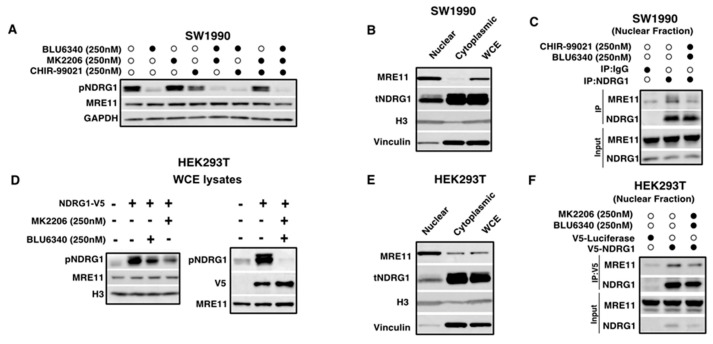
NDRG1-MRE11 complex formation is regulated by NDRG1 phosphorylation. (**A**) Western blot showing whole cell extracts (WCE) from SW1990 cells treated with SGK1 inhibitor (BLU6340), AKT inhibitor (MK2206) or GSK3 inhibitor (CHIR-99021) for 24 h alone and in combination and the inhibitor effects on p-NDRG1. The inhibitors impact was also assessed on total MRE11 protein levels. Black dots indicate presence of drug. (**B**) Western blot showing fractionated cell lysates from SW1990 cells. H3 and vinculin are shown as a marker for fractionation. (**C**) SW1990 nuclear fractions were used for endogenous co-immunoprecipitation between NDRG1 and MRE11 using NDRG1 antibody. IgG rabbit antibody was used as a negative control. Input shows total MRE11 and tNDRG1 levels in the lysate. (**D**) Western blot showing whole cell extracts (WCE) from HEK293T cells treated with SGK1 inhibitor (BLU6340) or AKT inhibitor (MK2206) for 24 h were probed for p-NDRG1 and MRE11 (**left** panel). WCE of HEK293T cells with V5-NDRG1 were treated with the combination of MK2206 and BLU6340 for 24 h (**right** panel) and probed for p-NDRG1. Plus signs indicate presence of drug. (**E**) Western blot showing fractionated cell lysates from HEK293T cells. H3 and vinculin are shown as a marker for fractionation. (**F**) HEK293T nuclear fractions from were used for endogenous co-immunoprecipitation using V5 antibody. V5-luciferase expressing cells were used as negative control for immunoprecipitation. The uncropped blots are shown in Supplementary Materials.

NDRG1 C-terminal phosphorylation is known to be regulated by various kinases including SGK1, AKT, and GSK3 [35,36,37]. The NDRG1 C-terminus contains several phosphorylation sites and a unique three-tandem repeat sequence of 10 amino acids (GTRSRSHTSE), incorporating multiple key phosphorylation sites [35]. SGK1 has been demonstrated to phosphorylate NDRG1 at several threonine and serine residues, specifically Thr238, Ser330, Thr346, Thr356, Thr366. Of these, Thr346, Thr356 and Thr366 are located within the three-tandem repeat region. SGK1-mediated phosphorylation has been shown to prime NDRG1 by converting it into a substrate for subsequent GSK3-mediated phosphorylation [35]. Following SGK1 priming, GSK3 can phosphorylate the residues Ser342, Ser352 and Ser362, all of which are located within the three-tandem repeat [35]. Additionally, GSK3-mediated phosphorylation of NDRG1 has been shown to regulate the phosphorylation of other NDRG1 phosphorylation residues [38]. AKT-mediated phosphorylation of NDRG1 is less well understood but has been observed to overlap with SGK1 phosphorylation sites. This overlap, however, appears to be cell-type-specific [37,39]. We first aimed to understand which of these kinases regulate the phosphorylation of the Thr346 site of NDRG1 in the SW1990 pancreatic cancer cell line. Treatment with the SGK1 inhibitor, BLU6340, resulted in the highest inhibition of NDRG1 phosphorylation, while treatment with the GSK3 inhibitor, CHIR-99021, also reduced the level of NDRG1 phosphorylation though to a lesser extent (Figure 3A). Treatment with the AKT inhibitor, MK2206, had no discernible effect on NDRG1 phosphorylation at this site, and none of the inhibitors had effect on MRE11 protein levels (Figure 3A). Based on these results, SGK1 and GSK3 are the primary kinases that regulate the phosphorylation of NDRG1 in the SW1990 cells. To examine how NDRG1 phosphorylation at Thr346 influences nuclear interaction or protein complex formation between MRE11 and NDRG1 in SW1990 cells, we treated SW1990 cells with the SGK1 inhibitor (BLU6340) and GSK3 inhibitor (CHIR-99021). We then fractionated the cell lysates to obtain the nuclear fraction (Figure 3B). Using this nuclear fraction, we performed endogenous co-IP by pulling down NDRG1 and immunoblotting for MRE11. Inhibition of SGK1- and GSK3-mediated phosphorylation of NDRG1 resulted in reduced complex formation between NDRG1 and MRE11 in the nucleus (Figure 3C). We were also interested in examining the role of NDRG1 phosphorylation on the NDRG1-MRE11 complex formation in HEK293T cells, a non PDAC cell line. In HEK293T cells, treatment with the AKT inhibitor (MK2206) resulted in the highest inhibition of NDRG1 phosphorylation, (Figure 3D), however the combination of AKT and SGK inhibition completely removed the NDRG1 phosphorylation (Figure 3D). This suggests that AKT and SGK1 function cooperatively as the primary kinases that regulate the phosphorylation of the NDRG1 Thr346 site in HEK293T cells. We then assessed the role of NDRG1 phosphorylation on the NDRG1-MRE11 complex formation in HEK293T cells using the nuclear fraction from cells treated with the combination of AKT inhibitor (MK2206) and SGK1 inhibitor (BLU6340) (Figure 3E). Similarly to our findings in SW1990, inhibition of NDRG1 phosphorylation resulted in reduced binding between NDRG1 and MRE11 in the nucleus (Figure 3F). Altogether, these data suggest that the phosphorylation of NDRG1 at Thr346 is critical regulatory post-translational modification that mediates its nuclear complex formation with MRE11.

### 3.4. In Vitro Studies Using Purified Proteins Suggest That the NDRG1-MRE11 Interaction Is Likely Indirect

**Figure 4:**
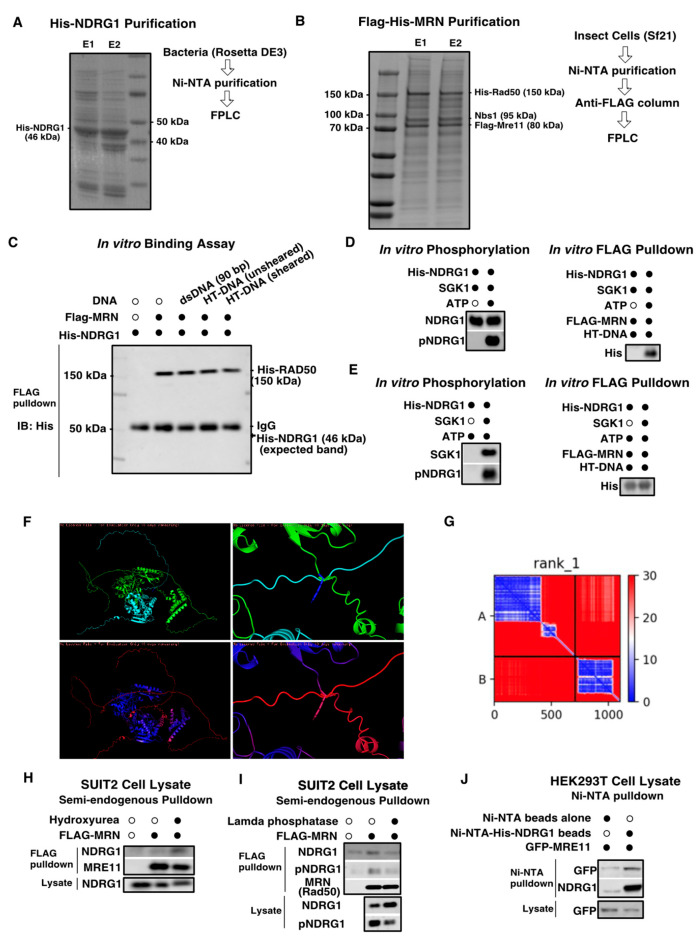
NDRG1-MRE11 interaction is likely indirect. (**A**) Rosetta (DE3) competent cells were transformed with pET-28a-NDRG1 plasmids to express His-tagged full length WT NDRG1. Soluble extract was incubated with Ni-NTA beads and his-tagged NDRG1 proteins were eluted. Eluted proteins were concentrated then loaded onto Superdex75 10/300 column for size exclusion chromatography. Elution fractions containing desired NDRG1 were collected, run on SDS-PAGE, and stained with Coomassie Brilliant Blue R-250. Lane 1 (E1): most pure fraction, lane 2 (E2): less pure fractions pooled. (**B**) Sf21 insect cells were used to generate WT MRN complex: FLAG-Mre11-WT, Nbs1, 6xHis-Rad50. Baculovirus for each individual member of MRN complex were generated and Sf21 cells were infected with all three baculoviruses to generate full MRN complex. Soluble extract from insect cells containing MRN complex was incubated with Ni-NTA beads and his-tagged MRN complex was eluted. Eluted MRN complex was concentrated and loaded onto Superose 6 column for size exclusion chromatography. Elution fractions containing desired MRN complex were collected, run on SDS-PAGE, and stained with Coomassie Brilliant Blue R-250. lane 1 (E1): most pure fraction, lane 2 (E2): less pure fraction. (**C**) In vitro binding assay using purified FLAG-MRN complex and purified His-NDRG1 in the presence of various DNA substrates including double stranded DNA (dsDNA) 90 bp, herring testes (HT) DNA sheared, HT-DNA unsheared. FLAG M2 resin was used to pulldown FLAG-MRN complex, and eluate was subjected to western blotting with His tag antibody. (**D**) In vitro pulldown with purified FLAG-MRN complex and purified His-NDRG1 after SGK1 in vitro phosphorylation of His-NDRG1. (Left) In vitro phosphorylation reaction of his-NDRG1 in the absence and presence of ATP. (Right) In vitro pulldown using products from in vitro phosphorylation reaction. Black dots indicate the presence of specified proteins/compounds. (**E**) In vitro pulldown with purified FLAG-MRN complex and purified His-NDRG1 after SGK1 in vitro phosphorylation of His-NDRG1. (Left) In vitro phosphorylation reaction of his-NDRG1 in the absence and presence of SGK1. (Right) In vitro pulldown using products from in vitro phosphorylation reaction. (**F**) AlphaFold does not predict high confidence direct binding between MRE11-NDRG1. AlphaFold2 predictions of the best NDRG1-MRE11 multimer model. Top: Colored by chain (green = MRE11, cyan = NDRG1), Bottom: Colored by pLDDT (blue = high confidence, red = low confidence). Right: Zoomed out, Left: Zoomed in. (**G**) Predicted aligned error from AlphaFold prediction of NDRG1-MRE11 multimer. (**H**) Semi-endogenous pulldown using SUIT2 lysate with in vitro purified FLAG-MRN complex. SUIT2 cells were either untreated or treated with 2 mM hydroxyurea overnight. Lysates from SUIT2 cells were incubated with in vitro purified FLAG-MRN complex and M2 Flag resin overnight and probed for NDRG1 and MRE11 by Western blotting. (**I**) Semi-endogenous pulldown using SUIT2 lysate with in vitro purified FLAG-MRN complex. SUIT2 cells were either untreated or treated with lambda phosphatase before incubating with the purified FLAG-MRN complex and M2 Flag resin overnight, and probed for phospho- and tNDRG1 by Western blotting. (**J**) Semi-endogenous pull-down using Ni-NTA beads that were pre-conjugated to in vitro purified His-NDRG1, and Ni-NTA-His-NDRG1 beads were added to HEK293T cell lysates expressing GFP-MRE11, after which the beads were probed for the presence of GFP-MRE11. The uncropped blots are shown in Supplementary Materials.

Our data suggest that NDRG1 and MRE11 are found in a same protein complex, however these experiments do not address whether there is a direct protein-protein binding between the two proteins. To investigate if MRE11-NDRG1 bind each other directly in vitro and to understand how this interaction affects the molecular functions of MRE11 and NDRG1, we first purified His-tagged NDRG1 from bacteria and FLAG-tagged MRN complex from insect cells (Figure 4A,B). These purified proteins were used for subsequent binding assays to determine whether the NDRG1 and MRE11 interact directly. We reasoned that the interaction may require the presence of DNA, so we performed a pulldown using the purified proteins in the presence of various DNA substrates, including a 90 bp dsDNA and herring testes (HT) DNA sheared to simulate damaged DNA. However, we did not detect any direct binding between the purified MRN complex and NDRG1 under any of the conditions tested (Figure 4C). Given our data showing that inhibition of NDRG1 phosphorylation reduced the endogenous interaction between NDRG1 and MRE11 (Figure 3) in cellular context, we next hypothesized that the phosphorylation of NDRG1 might be required to mediate the binding in vitro. To test this in vitro, we performed an in vitro phosphorylation reaction for NDRG1 using recombinant SGK1 in the presence or absence of ATP and then subjected the products to an in vitro FLAG-MRN pulldown. Interestingly, we observed the pulldown of His-NDRG1 only in the presence of ATP (Figure 4D). To ensure that the observed binding was due to NDRG1 phosphorylation and not an ATP-related artifact, we performed a control experiment where we repeated the in vitro phosphorylation reaction in the presence or absence of SGK1, with ATP present in both reactions. The products of the reactions were subjected to FLAG-MRN pulldown, and we observed that His-NDRG1 was pulled down with the FLAG-MRN complex in both conditions, even in the absence of SGK1 (Figure 4E). This result suggested that the ATP-dependent binding observed earlier was likely an experimental artifact caused by the presence of ATP on the affinity beads or the MRN complex itself.

To further investigate the potential for a direct interaction between MRE11 and NDRG1, we used the multimer structure prediction tool, AlphaFold2, to predict any putative binding interfaces. The highest-scoring model predicted binding between R402 of MRE11 and G339 of NDRG1 (Figure 4F). However, this predicted binding site is located within the unstructured regions of both proteins casting doubt to the accuracy of this prediction. Furthermore, the corresponding predicted Local Distance Difference Test (pLDDT) coloring shows low confidence scores (low pLDDT) across the putative binding region (Figure 4G). This low confidence AlphaFold2 model corroborates our observations using purified proteins, supporting the conclusion that a stable, direct interaction between MRE11 and NDRG1 is highly unlikely.

However, we hypothesized that the two proteins could still bind via a scaffolding protein, and binding would be present in a cell lysate. To confirm that our purified proteins can pull down the endogenous binding partner from a cell lysate, the FLAG-MRN complex was used to pull down endogenous NDRG1 from the Suit2 PDAC cell lysate. These data show that the FLAG-MRN pulls down NDRG1 particularly in the presence of hydroxyurea (HU) a DNA damaging and fork stalling agent (Figure 4H). Additionally, treatment of the lysate with lambda phosphatase to remove NDRG1 phosphorylation, reduced the complex formation, confirming our prior data showing that NDRG1 phosphorylation is important for the binding (Figure 4I). We also confirmed that the recombinant His-NDRG1 was able to pull down GFP-tagged MRE11 in HEK293T cells (Figure 4J). Overall these results strongly suggest that MRE11 and NDRG1 do not bind each other directly but rather through a protein complex.

### 3.5. MRE11 Exonuclease Activity and NDRG1 Phosphorylation Act Together to Protect Nascent DNA at Stalled Forks

**Figure 5:**
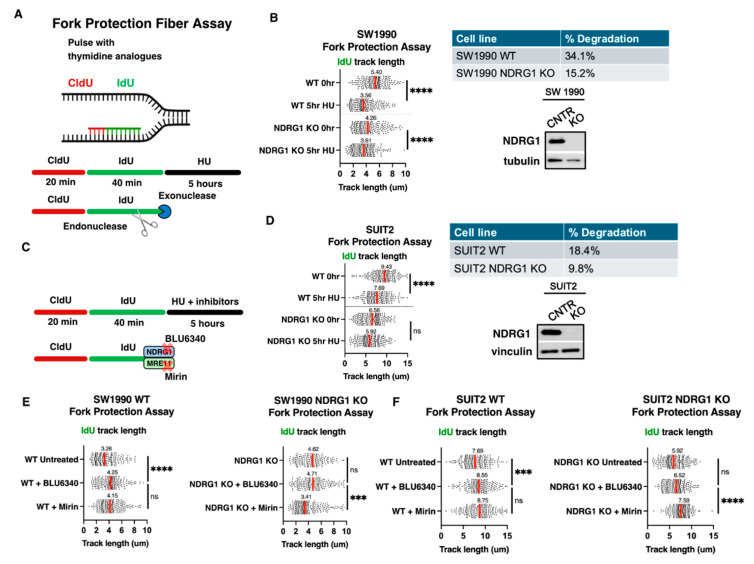
MRE11 exonuclease activity and NDRG1 phosphorylation contributes to the degradation of nascent DNA at stalled forks but is cell type specific. (**A**) Schematic showing the experimental plan for fork protection DNA fiber assays. Cells were pulsed with CldU for 30 min, followed by IdU for 30 min. Then cells were pulsed with 2 mM HU for 5 h. IdU track lengths were measured to assess the level of fork protection or degradation at stalled forks. (**B**) (**left**) Fork protection assay was performed in SW1990 WT and NDRG1 KO cells. Samples were taken before and after the 5 h HU treatment to establish the baseline level of nascent DNA degradation in SW1990 cells. IdU track lengths were quantified for ≥200 double-labelled fibers. Table on the **right** showing the calculated percentage of degradation observed during 5 h HU treatment in SW1990 WT and NDRG1 KO cell lines. (**C**) Schematic showing the experimental plan for fork protection DNA fiber assay in the presence of mirin to inhibit MRE11 exonuclease activity, and BLU6340 to inhibit NDRG1 phosphorylation. (**D**) Fork protection assay in SW1990 WT cells in the presence of 250 nM BLU6340 or 30 uM mirin. IdU track lengths were quantified for ≥200 double-labelled fibers. Table on the **right** showing the calculated percentage of degradation observed during 5 h HU treatment in SUIT2 WT and NDRG1 KO cell lines. (**E**) Fork protection assay in SW1990 WT and NDRG1 KO cells in the presence of 250 nM BLU6340 or 30 uM mirin. IdU track lengths were quantified for ≥180 double-labelled fibers. (**F**) Fork protection assay in SUIT2 WT and NDRG1 KO cells in the presence of 250 nM BLU6340 or 30 uM mirin. IdU track lengths were quantified for ≥180 double-labelled fibers. For all fork protection DNA fiber experiments, Mann–Whitney test was applied to determine significance. **** *p* < 0.0001, *** *p* < 0.001, n.s.: non-significant. Red lines in all figures represents median track lengths. Pooled data from at least N = 2 biological replicates are shown. The uncropped blots are shown in Supplementary Materials.

Our previous work demonstrated that NDRG1 was important for replication fork progression and stalled fork recovery. Additionally, MRE11 is known to play a role in stalled fork recovery by nucleolytically degrading reversed forks after fork stalling [12,14]. To gain more mechanistic insight into how the MRE11-NDRG1 complex may be functioning at the replication fork, we performed the fork protection DNA fiber assay, which allows us to assess the degradation or protection of nascent DNA at stalled replication forks (Figure 5A). First, we established the baseline level of nascent DNA degradation at stalled forks in SW1990 WT and NDRG1 KO cells by comparing the IdU track lengths before and after the 5 h hydroxyurea treatment. SW1990 WT cells underwent proportionally higher levels of nascent DNA degradation compared to NDRG1 KO cells (34.1% vs. 15.2%, respectively) (Figure 5B), similar data was observed in Suit2 cells (Figure 5D). Next, we performed the fork protection assays in the presence of SGK1 inhibitor (BLU6340) to abolish NDRG1 phosphorylation, and mirin to inhibit MRE11 exonuclease activity, to determine whether NDRG1 phosphorylation and/or MRE11 exonuclease activity were involved in degrading or protecting nascent DNA at stalled forks in SW1990 cells (Figure 5C). In SW1990 WT cells, mirin and BLU6340 treatment both protected nascent DNA at stalled forks from degradation suggesting that both MRE11 exonuclease activity and NDRG1 phosphorylation promote the degradation of nascent DNA at stalled forks (Figure 5E). To determine whether NDRG1 and MRE11 function in the same pathway to degrade nascent DNA at stalled forks, we performed the fork protection assay in NDRG1 KO cells. When NDRG1 KO cells were treated with BLU6340, we no longer observed fork protection suggesting that the effects of BLU6340 in fork degradation were specific to SGK’s kinase activity on NDRG1 phosphorylation rather than its effects on its other targets (Figure 5E). Surprisingly, treatment with mirin did not result in fork protection in NDRG1 KO cells, suggesting that MRE11 may act in the same pathway to degrade nascent DNA at stalled forks, and that MRE11 might depend on NDRG1 since in the absence of NDRG1, inhibition of MRE11 exonuclease activity by mirin no longer had a fork protective effect (Figure 5E). The functional relationship of MRE11 exonuclease activity and NDRG1 phosphorylation in stalled fork dynamics do seem to be cell type specific. When we performed the same experiments in SUIT2 cells, mirin treatment resulted in fork protection in NDRG1 KO cells suggesting that the MRE11-dependent fork degradation is not dependent on NDRG1 in SUIT2 cells, and that there could be cell type specificity for these functions of NDRG1 and MRE11 in fork degradation (Figure 5F).

## 4. Discussion

We previously identified NDRG1 as a DNA repair protein that influences chemoresistance to fork-stalling drugs in pancreatic cancer [5]. In the current study, we aimed to further elucidate the mechanistic details regarding NDRG1’s role in DNA repair and replication by identifying and characterizing novel NDRG1 binding partners. Using BioID, we identified MRE11 in the same complex as NDRG1. We found that the NDRG1-MRE11 complex formation was enriched during late S/early G2 phases of the cell cycle, and upon treatment with HU suggesting that the protein complex is temporally and functionally coupled to the replication stress response. Furthermore, our data show that the interaction is regulated by NDRG1 phosphorylation, as inhibition of NDRG1 phosphorylation resulted in weakened complex formation between NDRG1 and MRE11. Given that the purified proteins failed to interact in vitro, we concluded that the interaction is likely indirect and mediated by a scaffolding protein or additional post-translational modifications (PTM). Interestingly, we observed that MRE11 exonuclease activity and NDRG1 phosphorylation both contribute to the degradation of nascent DNA at stalled forks, and that they may act in the same pathway to carry out this DNA resection function in a cell-type specific manner.

MRE11 is known to play an important role in the cellular response to replication stress and fork stalling, and this role has been attributed to nuclease-dependent and nuclease-independent functions of MRE11. Consistent with this, MRE11 has recently been identified as a novel player in R-loop tolerance [40]. Specifically, Chang et al. showed that depletion of the MRN complex resulted in R-loop accumulation and impaired fork progression, which was attributed to increased transcription-replication conflicts (TRCs) [40]. This observation of MRE11 aligns nicely with our prior findings that loss of NDRG1 or inhibition of SGK1-mediated NDRG1 phosphorylation resulted in replication fork stalling, increased R-loop burden, transcription-replication conflicts, and higher sensitivity to chemotherapies that cause increase in R-loops [5]. The exact mechanism by which NDRG1 facilitates replication fork protection by reducing R-loop burden remains unknown. Interestingly, our fork protection fiber assays in the current study revealed a novel function of NDRG1 at stalled forks. We found that the inhibition of NDRG1 as well as NDRG1 KO cells experience less degradation of nascent DNA at stalled forks, suggesting that NDRG1 and its phosphorylation are involved in promoting the degradation of nascent DNA at stalled forks (Figure 5). The main players that have been shown to mediate R-loop resolution include RNA nucleases like RNase H1 and H2, which target and degrade the RNA strand within RNA:DNA hybrids, and DNA helicases like Senataxin (SETX), which unwind the RNA-DNA hybrid [41,42]. Recent studies have also implicated some DNA-targeting nucleases as novel R-loop processing proteins. For example, the MUS81 endonuclease promotes R-loop suppression via activation of the ATR-Chk1 pathway, and DNA2-mediated resection of MUS81-cleaved replication forks promotes fork restart after R-loop-mediated replication stress [43,44]. Since we observe that both NDRG1 phosphorylation and MRE11 exonuclease activity promote the degradation of nascent DNA at stalled forks, we hypothesize that this function may be mechanistically related to their role in R-loop resolution. It is important to note that the study by Chang et al. reported that MRE11’s role in R-loop resolution was independent of its nuclease activity in HeLa and TK6 cells [40]. However, given the context- and cell-type-specific nature of DDR factors, we hypothesize that in certain cell types and cancer contexts, such as pancreatic cancer, MRE11 exonuclease activity and phospho-NDRG1 could potentially act together to play a novel nuclease-dependent role in resolving R-loop-mediated fork stalling. Therefore, future studies should focus on investigating whether the MRE11- and NDRG1-dependent fork degradation we observed is directly linked to the resolution of R-loops. We did observe cell-type specific differences in the stalled fork degradation phenotype between two different pancreatic cancer cell lines used in our study, SW1990 and SUIT2. These different responses may reflect underlying differences in cellular state and stress response pathways. For example, SUIT2 cells have been characterized to be more mesenchymal and aggressive, with high metastatic potential, suggesting a distinct transcriptional and epigenetic landscape compared to SW1990 cells. These types of differences in cell state may be associated with altered responses to replication stress and fork stalling. Furthermore, in our recent study investigating the role of a novel interaction between NDRG1 and TGM2 in DNA replication and repair, we observed that the interaction was regulated in a cell type-specific manner, further pointing to the highly pleiotropic nature of NDRG1 and its interactions with other proteins [11].

Although we detected the MRE11-NDRG1 binding in cellular context (Figure 1), the absence of binding in vitro between the purified proteins (Figure 4) suggests that the interaction is indirect. This requirement for a cellular environment implies that crucial scaffolding protein(s) or a specific PTM may be required to bridge the interaction. This is highly plausible, given that both NDRG1 and MRE11 are known to undergo extensive PTMs to regulate and fine-tune their diverse functions [45]. Furthermore, we identified other interesting putative NDRG1 binding partners in our BioID screen including SAMHD1 (SAM and HD domain-containing protein 1) (Figure 1D), which has been previously shown to interact with MRE11, and this protein-protein interaction was shown to promote the degradation of nascent DNA at stalled forks [46]. It is possible that MRE11 and NDRG1 may form a complex with proteins such as SAMHD1, or other proteins involved in stalled fork processing to exert their effects on DNA replication and repair. Uncovering the exact factor(s) required for this interaction is critical, as it would reveal essential regulatory mechanisms for MRE11 or NDRG1 at stalled forks. Future studies should focus on identifying the minimal components required to reconstitute the interaction in vitro.

## 5. Conclusions

In summary, this study identifies and characterizes a novel protein-protein interaction between NDRG1 and MRE11, demonstrating that this association is regulated by NDRG1 phosphorylation and potentially dictates the function of both proteins in promoting the degradation of nascent DNA at stalled replication forks.

## Data Availability

All data presented in this manuscript are freely available upon request.

## Supplementary Materials

The following supporting information can be downloaded at: https://www.mdpi.com/article/10.3390/cancers18081303/s1. Supplementary File S1: Full list of BioID hits; Supplementary File S2: Uncropped Western blots.

## Abbreviations

The following abbreviations are used in this manuscript:

AKT	protein kinase B
CAF	cancer associated fibroblast
DSB	double-stranded breaks
DDR	DNA damage response
GSK3	glycogen synthase kinase
HR	homologous recombination
HU	hydroxyurea
IC50	inhibitory concentration 50
MRE11	meiotic recombination 11
MRN	MRE11-RAD50-NBS1
NDRG1	N-myc downstream regulated gene 1
PARP	poly ADP ribose polymerase
PDAC	pancreatic ductal adenocarcinoma
pLDDT	predicted Local Distance Difference Test
PLA	proximity ligation assay
SGK1	serum and glucocorticoid activated kinase 1
