# Supplementary material for: Interaction of NDRG1 and MRE11 Modulates DNA Replication and Repair
Source: Cancers (Basel). 2026 Apr 20;18(8):1303. doi: 10.3390/cancers18081303 (PMC13115477; doi:10.3390/cancers18081303)

Figure 1C

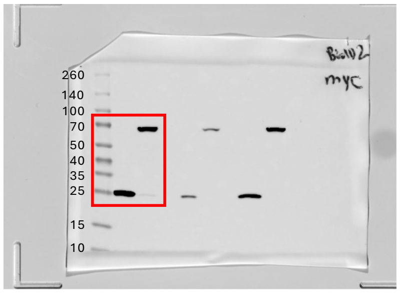

173:146

Myc-tag

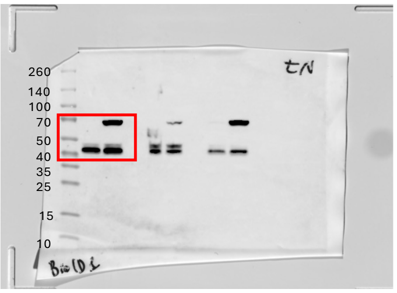

0:100

145:154

NDRG1

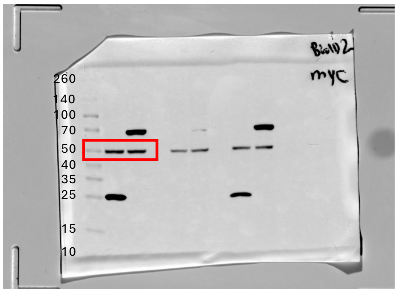

156:157

Tubulin

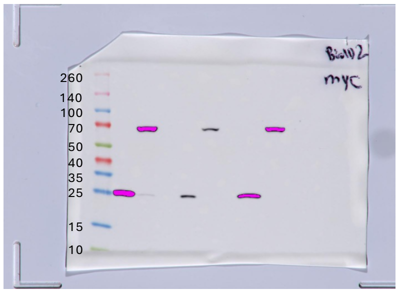

Figure 1 source WB

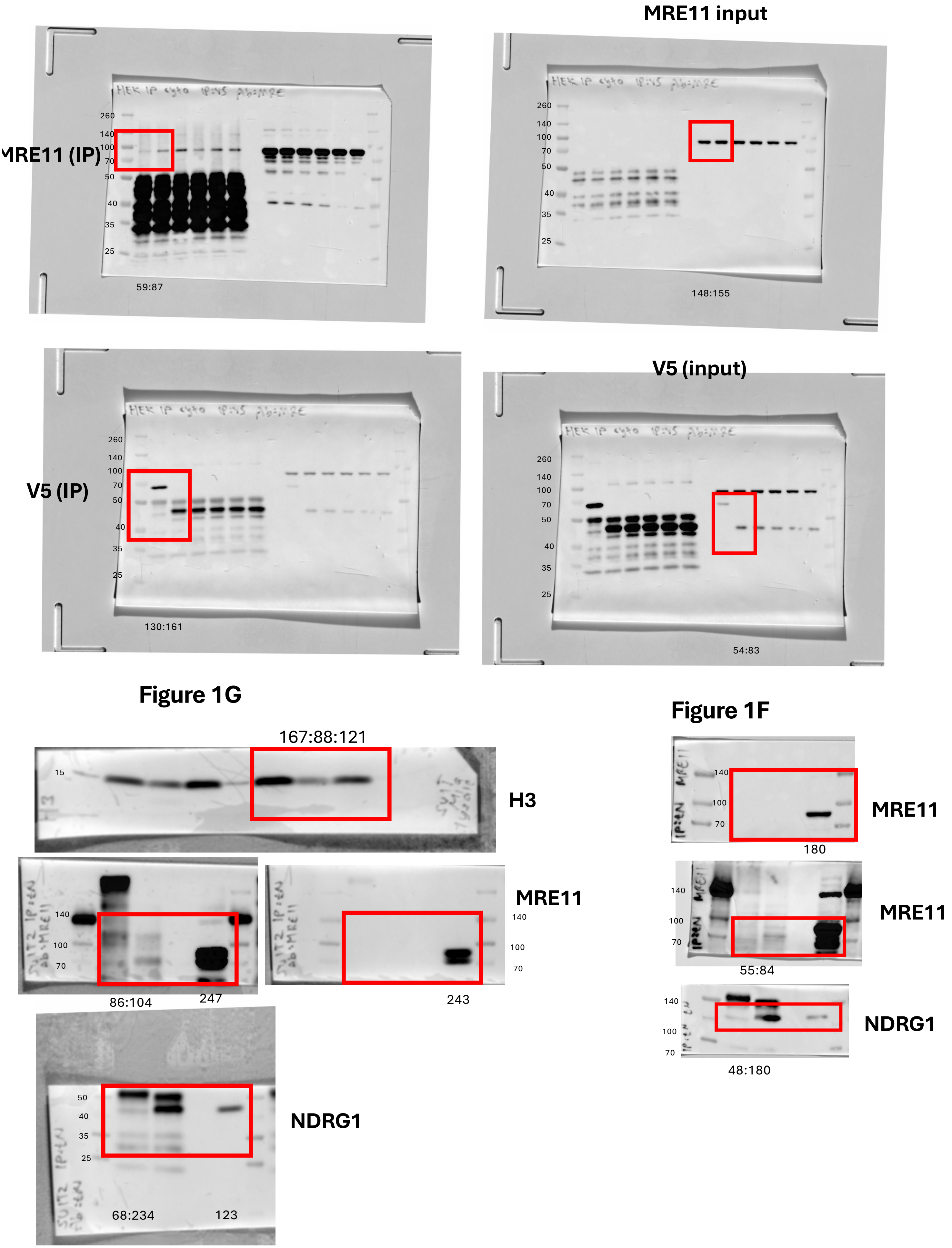

Figure 2 source WB

Figure 2A

Cyclin A2

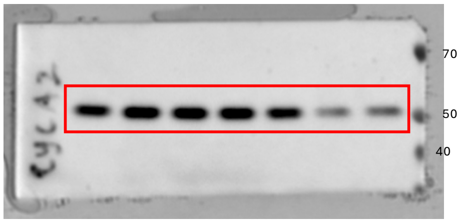

140:165:160:159:139:79:89

pCDK2

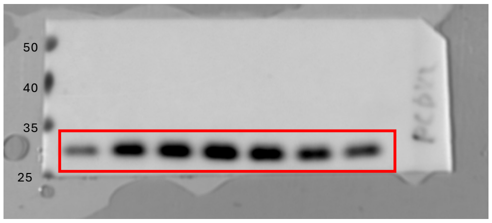

90:136:148:158:147:128:114

Vinculin

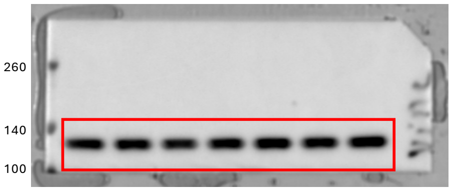

155:158:142:156:167:157:172

Figure 3 source WB

Figure 3A

pNDRG1

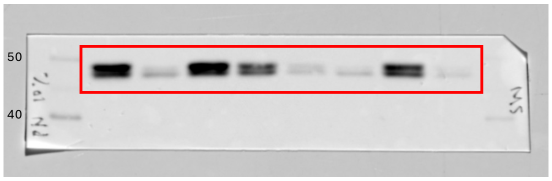

158:53:162:107:46:44:132:40

MRE11

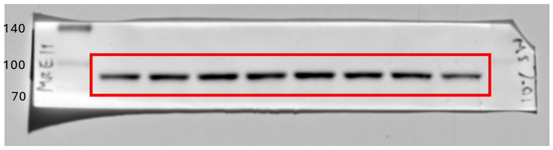

99:116:127:123:131:119:118:93

GAPDH

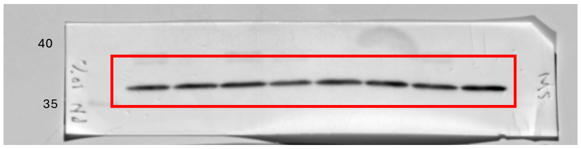

125:134:137:115:134:137:132:160

Figure 3 source WB

Figure 3D

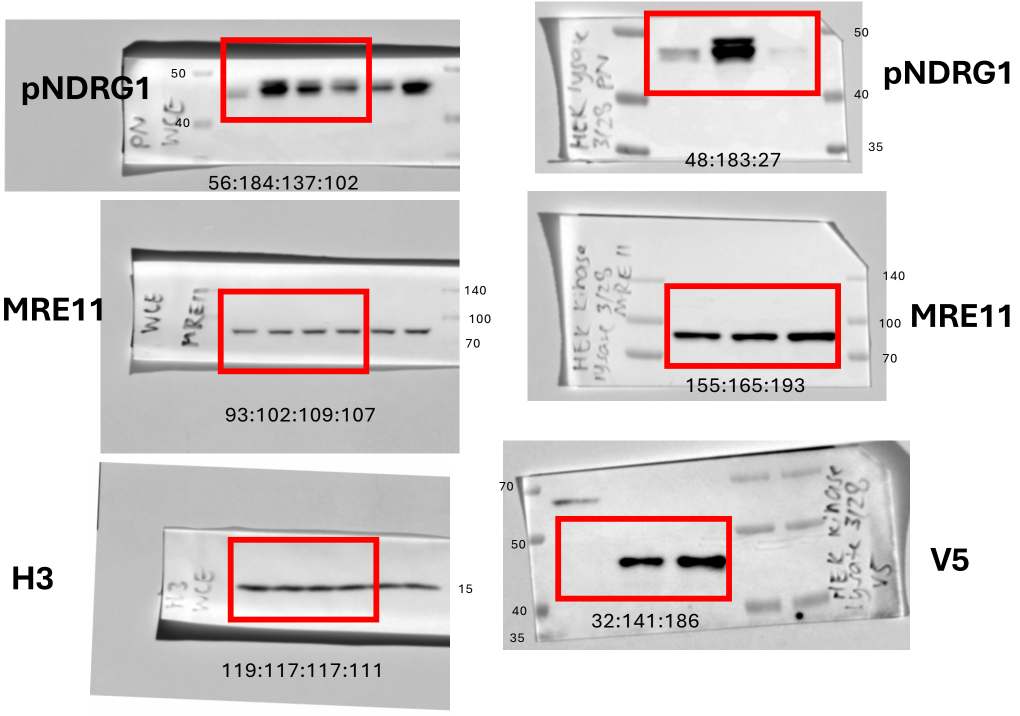

Figure 3C

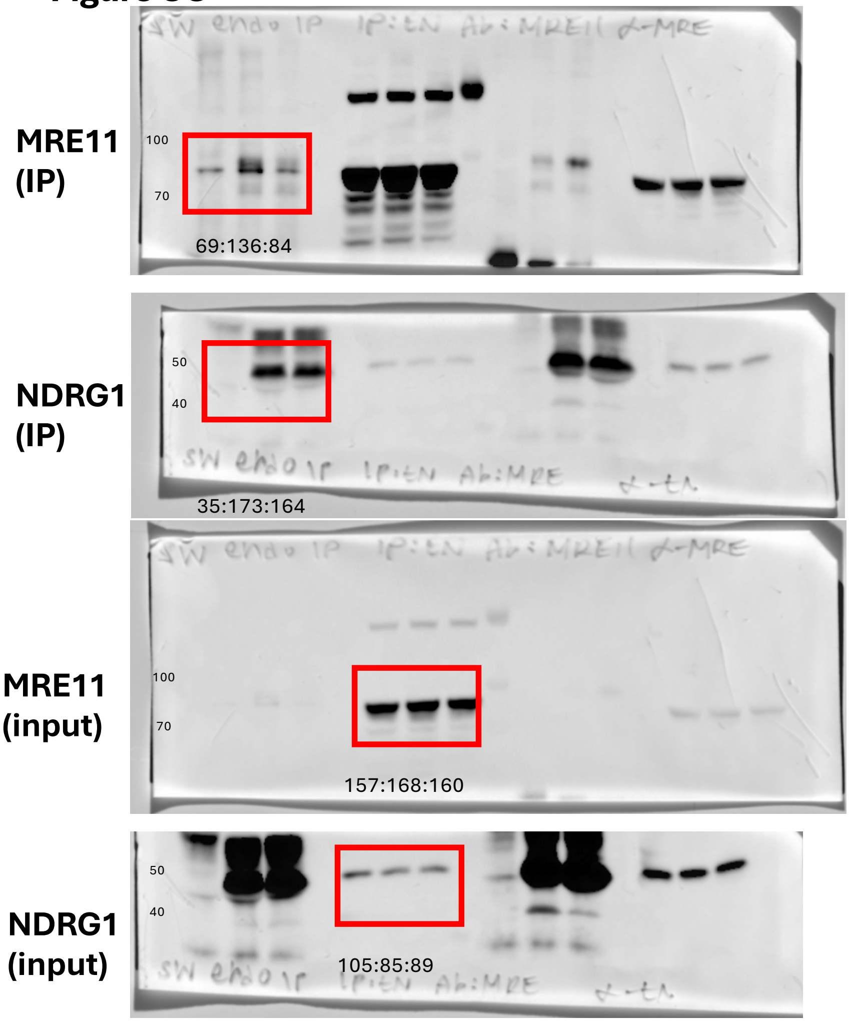

Figure 3F

MRE11 (IP, long exposure)

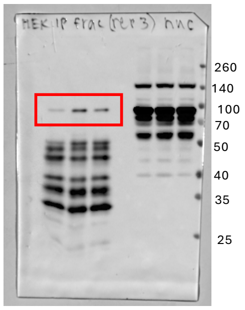

65:117:93

NDRG1 (IP)

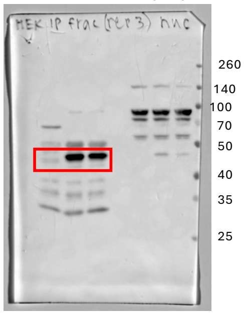

61:211:192

MRE11 (lysate, short exposure)

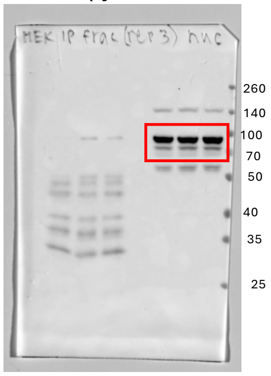

211:210:216

NDRG1 (lysate, long exposure)

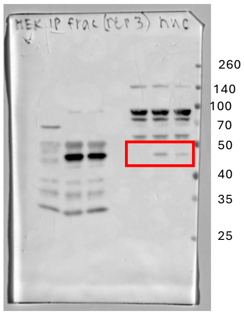

48:76:59

Figure 3B/E

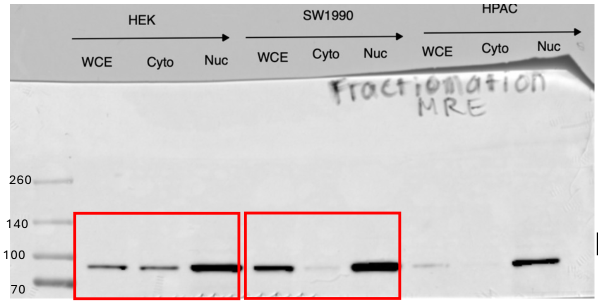

MRE11

85:78:178
135:37:233

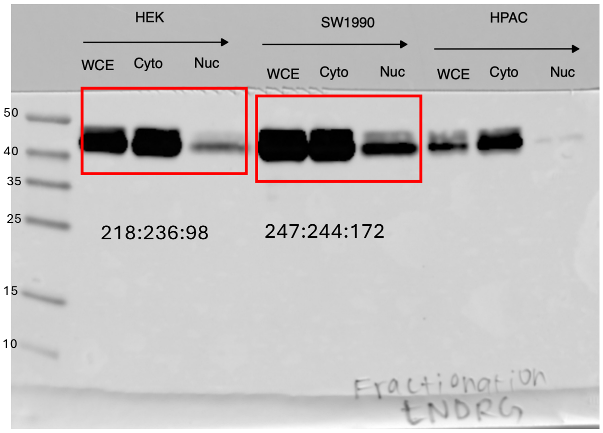

NDRG1

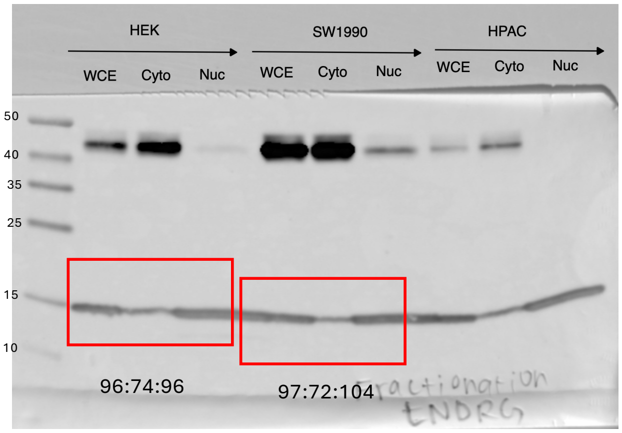

H3

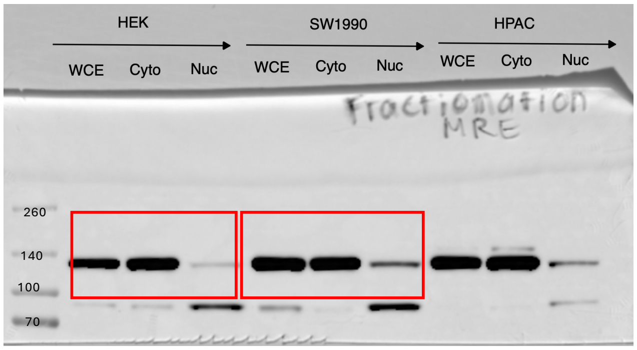

vinculin

189:215:52
230:223:95

Figure 4C

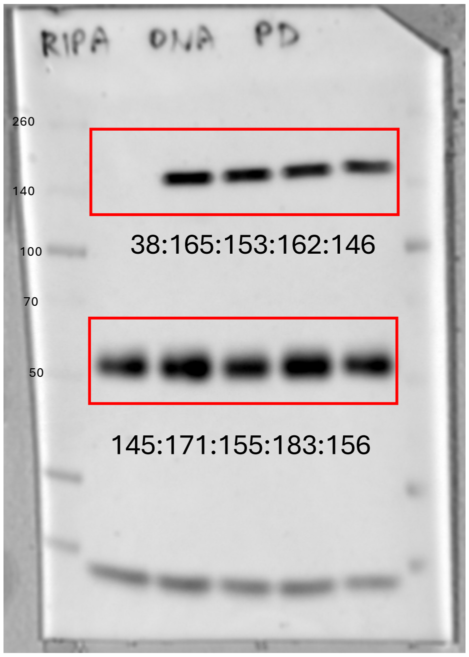

Figure 4D

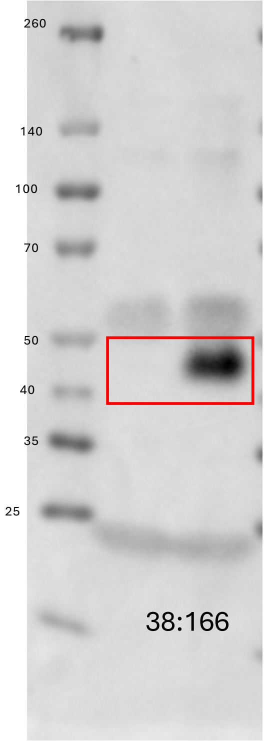

His

NDRG1

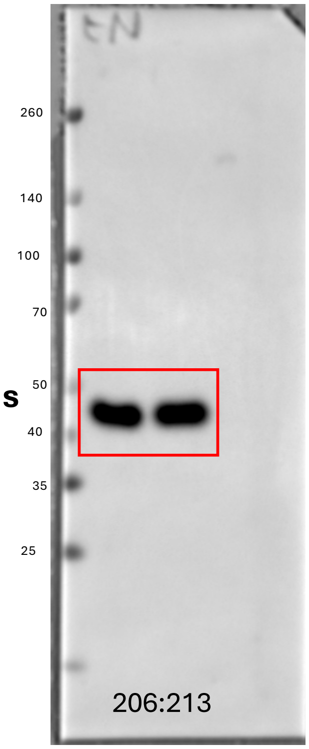

pNDRG1

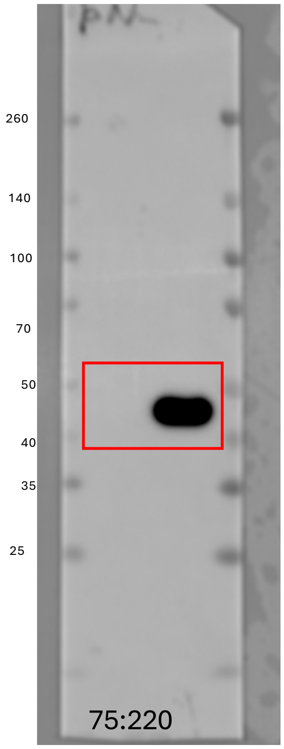

Figure 4E

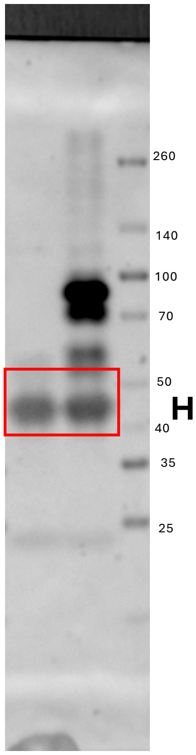

His

128:142

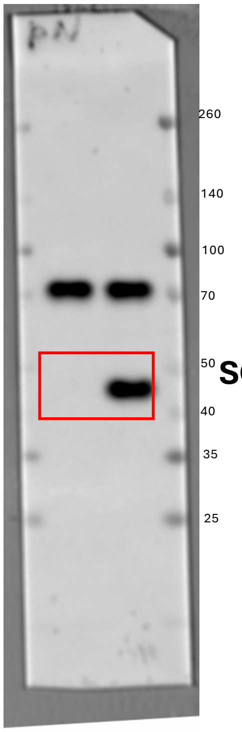

SGK

50:169

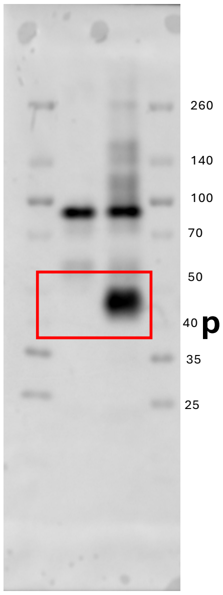

pNDRG1

47:175

Figure 4 source WB

Fig 4A Coomassie staining

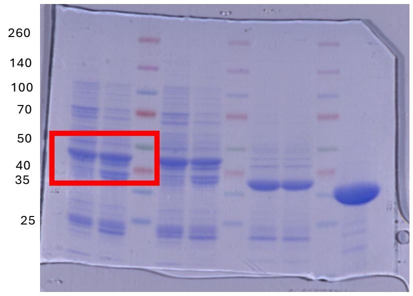

119:124

Fig 4B Silver staining

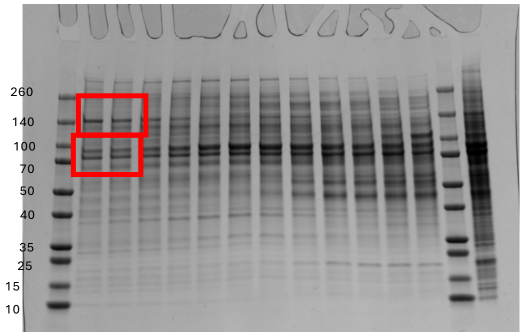

166:164
163:163

Figure 4I

NDRG1 pNDRG1 His-RAD50 (MRN) NDRG1 pNDRG1

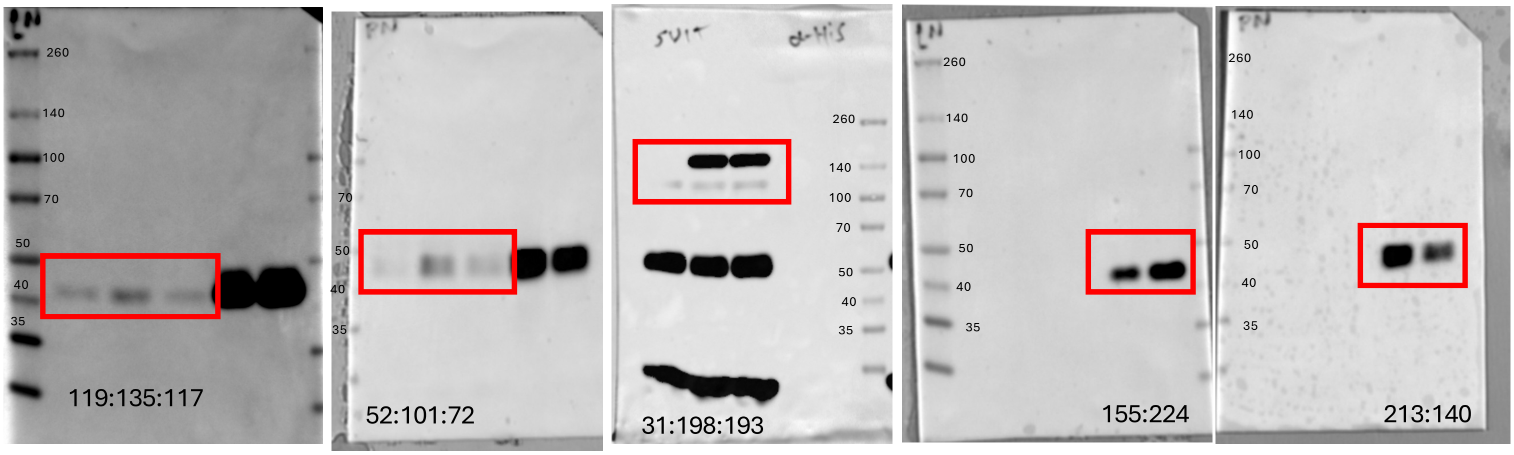

Figure 4H

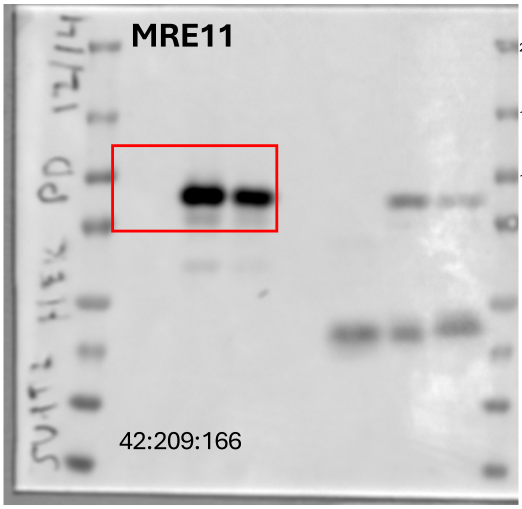

NDRG1

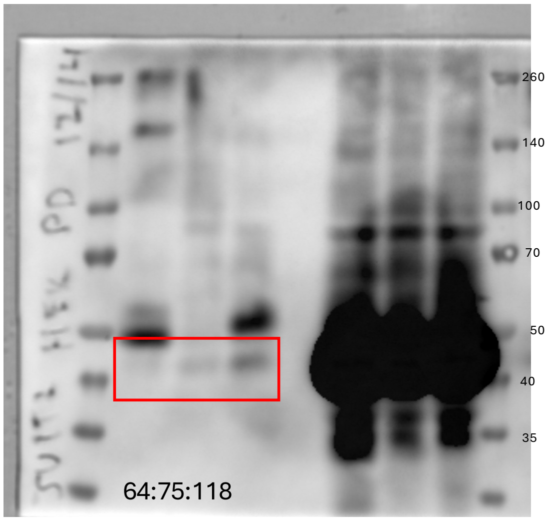

NDRG1 (input)

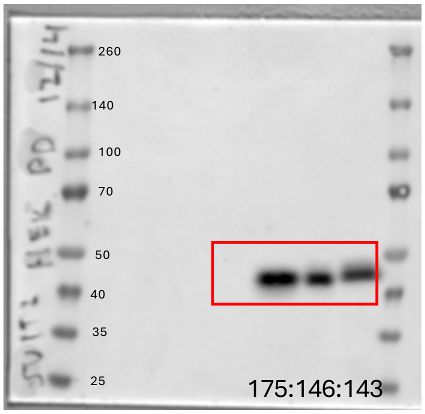

Figure 4J

GFP

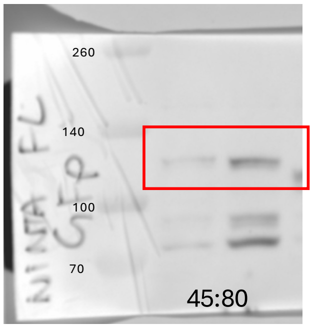

NDRG1

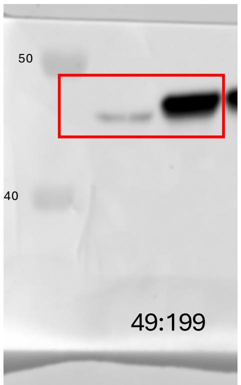

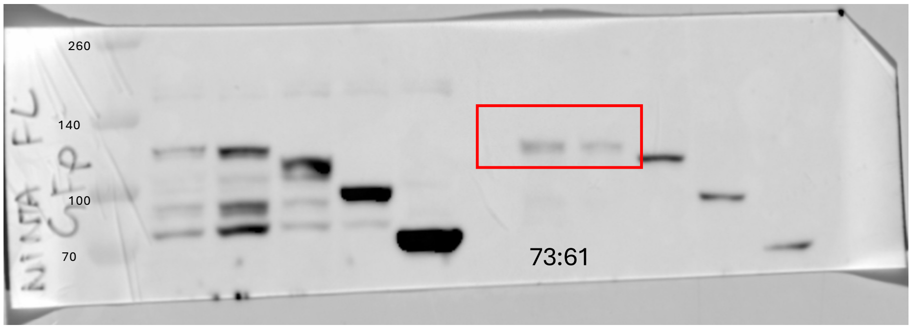

GFP (input)

Figure 5

Figure 5B

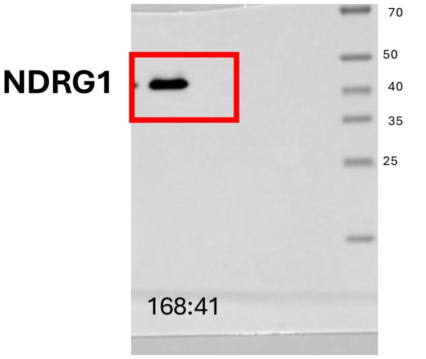

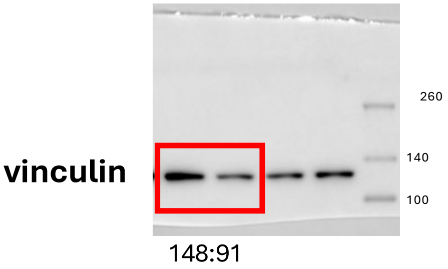

Figure 5D

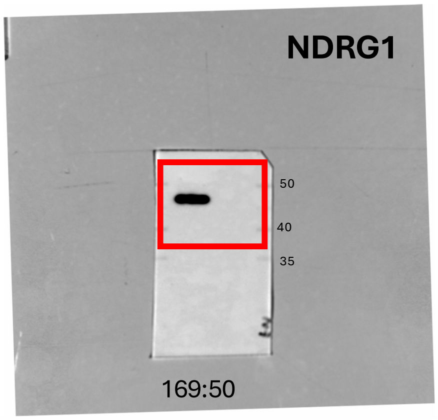

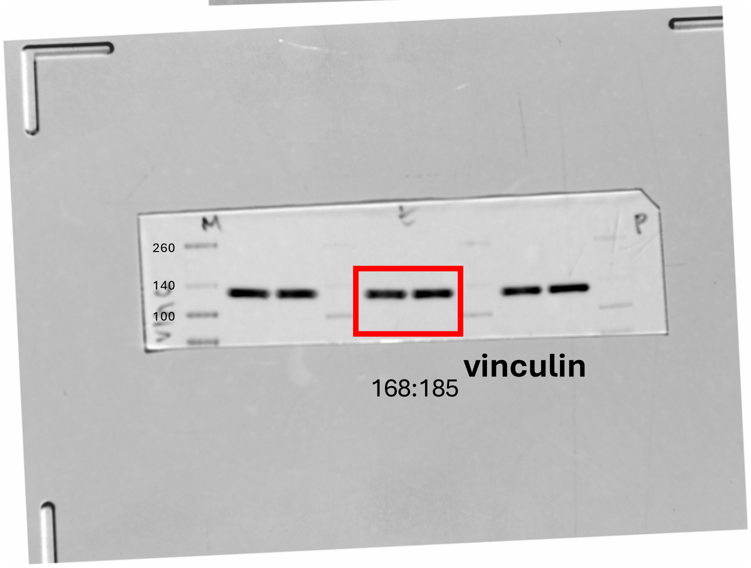

Supplement: Supplementary file 1 [file cancers-18-01303-s001.zip › cancers-4224136 supp 4.8/WB_source_data_HD_updated.pdf]
